# Ensuring *Emerging Themes in Epidemiology*’s continued success

**DOI:** 10.1186/s12982-021-00092-6

**Published:** 2021-01-26

**Authors:** Steven S. Coughlin

**Affiliations:** 1 Department of Population Health Sciences, Medical College of Georgia, Augusta University, 1120 15th Street, AE-1042, Augusta, GA 30912 USA; 2 Institute of Public and Preventive Health, Augusta University, Augusta, GA USA

As the new Editor-in-Chief of *Emerging Themes in Epidemiology*, I would like to take this opportunity to thank the previous Editor-in-Chief of the journal, Dr. Clarence Tam, as well as the current members of the Editorial Board and reviewers for their many contributions to the journal. The reviewers and Editorial Board members have important roles in ensuring the quality of the peer review process and the overall success of the journal.

Emerging Themes in Epidemiology is an open access, peer-reviewed journal that publishes original research papers to promote debate and discussion on practical and theoretical aspects of epidemiology. We welcome primary research and discursive articles. We particularly encourage submissions in the following areas:

- New epidemiological concepts and methods
- Novel ways of presenting and providing insights into existing epidemiological concepts and methods, including in a teaching environment
- Use of epidemiological research methods in non-medical settings
- Causal inference in epidemiology
- Applications of new technologies in field studies (e.g. mobile technologies, pattern recognition techniques, social networking tools)
- Ethical issues in epidemiological research
- New statistical methods, or novel uses of existing methods, in epidemiological studies
- Methodological developments in molecular and genetic epidemiology and novel applications in these areas
- Historical articles and re-assessments of classic papers

The journal considers the following types of article for publication: research papers, analytic perspectives, commentaries, debate articles, hypotheses, methodology articles, and reviews.

The articles published in the journal encompass virtually all epidemiology content areas and sub-disciplines including chronic disease epidemiology, infectious disease epidemiology, genetic epidemiology, occupational and environmental epidemiology, and public health practice. Due in part to the journal’s global readership and contributors, published articles also address important topics in global health.

To ensure the continued success and growth of the journal over time, it is important for reviewers and Editorial Board members to offer their recommendations and critiques in a timely fashion and for the journal to strive to reach decisions about publications in a reasonable timeframe. The continued success of the journal depends upon the timeliness of editorial decisions and the thoughtful contributions of many reviewers, as well as the productivity and insights of contributing authors. I look forward to contributing to the journal’s continued success and reading the many articles submitted to the journal for consideration for publication.

## Data Availability

Not applicable.

